# Fracture Resistance of Zirconia Abutments with or without a Titanium Base: An In Vitro Study for Tapered Conical Connection Implants

**DOI:** 10.3390/ma15010364

**Published:** 2022-01-05

**Authors:** Shota Watanabe, Tamaki Nakano, Shinji Ono, Yasufumi Yamanishi, Takashi Matsuoka, Shoichi Ishigaki

**Affiliations:** Department of Fixed Prosthodontics, Osaka University Graduate School of Dentistry, 1–8 Yamadaoka, Suita, Osaka 565-0871, Japan; watanabe.shota.dent@osaka-u.ac.jp (S.W.); onoshin@dent.osaka-u.ac.jp (S.O.); y-yamani@dent.osaka-u.ac.jp (Y.Y.); matsuoka.takashi.dent@osaka-u.ac.jp (T.M.); ishigaki.shoichi.dent@osaka-u.ac.jp (S.I.)

**Keywords:** zirconia abutment, titanium base, tapered conical connection, static load

## Abstract

Dental implants with tapered conical connections are often combined with zirconia abutments for esthetics; however, the effect of the titanium base on the implant components remains unclear. This study evaluated the effects of a titanium base on the fracture resistance of zirconia abutments and damage to the tapered conical connection implants. Zirconia (Z) and titanium base zirconia (ZT) abutments were fastened to Nobel Biocare (NB) implants and Straumann (ST) implants and subjected to static load testing according to ISO 14801:2016. The experiments were performed with 3 mm of the platform exposed (P3) and no platform exposed (P0). The fracture loads were statistically greater in the titanium base abutments than the zirconia abutments for the NB and ST specimens in the P0 condition. In the P3 condition of the ST specimens, the deformation volume of the ZT group was significantly greater than the Z group. The titanium base increased the fracture resistance of the zirconia abutments. Additionally, the titanium base caused more deformation in the P3 condition. The implant joint design may also affect the amount of damage to the implants when under a load. The mechanical properties of the abutment should be considered when selecting a clinical design.

## 1. Introduction

In dental implant treatments, especially in the esthetic zone, zirconia abutments are desired because of their superior esthetics and biocompatibility [1,2]. Currently used zirconia abutments are classified according to differences in the implant–abutment connection, and there have been various comparative studies on the mechanical properties of zirconia abutments in conventional connection modes (external butt joints and internal butt joints) [3,4].

A further classification was recently proposed to distinguish between two-piece abutments with a titanium base in the connecting part and one-piece abutments comprised entirely of zirconia [5]. Several studies have compared the mechanical properties of both types.

Several in vitro studies have reported that titanium base zirconia abutments were significantly more resistant to a bending moment than zirconia abutments [2,6,7,8,9,10]. Furthermore, the titanium base type is advantageous from a mechanical viewpoint because the titanium in the connecting part is likely to cause less wear in the implant body [8,11,12].

However, these mechanical studies often use different implant bodies [2,6,7,8,9,10] or compare samples produced by different computer-aided design/computer-aided manufacturing (CAD/CAM) systems [2,6,7,8,9,10]. How the inclusion of a titanium base affects the mechanical properties of zirconia abutments has not been evaluated using actual products.

When the titanium base is interposed in the connecting portion from the implant body, the component thickness may increase, making it difficult to maintain sufficiently thick surrounding tissue. Additionally, titanium bases tend to affect the color in thin gingiva [2,4,5,6,7,10,11,12,13,14,15]. Therefore, the esthetics of a titanium base are disadvantageous; however, there is no consensus on using titanium-based zirconia abutments.

Abutments for tapered conical connection implant bodies with platform shifting can prevent bone resorption and soft tissue retraction around the implant body [16,17,18]. However, clinical research has revealed problems with the fracture of zirconia abutments inside the implant body [9,15]. A low incidence of chipping and detachment of zirconia with titanium base abutments has also been reported [9]. There is a need for clarifying the effect on fracture resistance of different connection types of zirconia abutments in tapered conical connection implant bodies.

This study aimed to clarify the effect of a titanium base on the fracture strength of the abutment for the same implant with a conical connection. In addition, by observing the amount of deformation of the implant body, especially in bone resorption, the magnitude of harmful stress applied to the implant body until the abutment fracture was evaluated.

The null hypothesis were no differences in the fracture load or implant deformation between the titanium base and full zirconia abutments.

## 2. Materials and Methods

**Figure 1:**
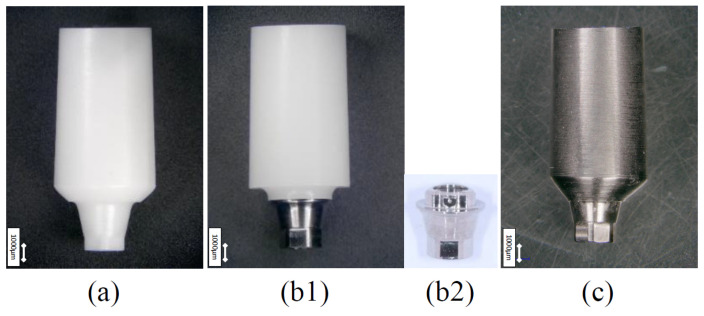
Test zirconia abutment specimens and controls (NB; Nobel Procera^®^). (**a**) Z-NB group (full zirconia type). (**b1**) ZT-NB group (titanium base type). (**b2**) genuine titanium base. The same one inserted into the zirconia in (**b1**). (**c**) T-NB group (titanium abutment).

**Figure 2:**
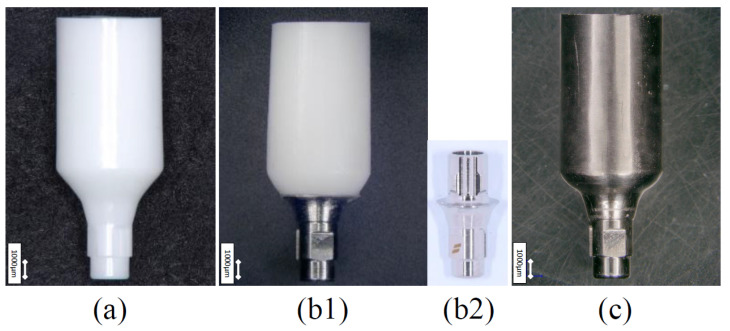
Test zirconia abutment specimens and controls (ST; CARES^®^). (**a**) Z-ST group (full zirconia type). (**b1**) ZT-ST group (titanium base type). (**b2**) genuine titanium base. The same one inserted into the zirconia in (**b1**). (**c**) T-ST group (titanium abutment).

Implant bodies from Nobel Biocare (NB) and Straumann (ST), which possess their own CAD/CAM systems and options to select the inclusion of a titanium base for zirconia abutment with the same taper joint body, were selected. Actual (i.e, not computer-aided) products were used for all relevant components. The implant bodies were Nobel Replace CC implants (NB:φ4.3 mm, 10 mm length, Nobel Biocare, Kloten, Switzerland) and Roxolid BLT implants (ST:φ4.1 mm, 10 mm length, Straumann, Basel, Switzerland). For the test sample abutments, two types of abutments (Z: full zirconia, ZT: titanium based) were prepared for both implant bodies (Z-NB, ZT-NB, Z-ST, and ZT-ST). Titanium abutments were used as controls (T-NB and T-ST) (Figure 1 and Figure 2). The ZT-NB group was fixed by mechanical engagement and co-clamping with an abutment screw to the zirconia and titanium base. In the ZT-ST group, the zirconia and titanium bases were fixed with adhesive resin cement (Resicem, Shofu, Kyoto, Japan). These differences in the fixing methods were as recommended by the manufacturer.

Test conditions were modified for this experiment according to ISO 14801:2016 [19], which is the standard for dynamic fatigue tests of dental endosseous implants. In particular, the platform of the implant body was placed 0 mm (P0) or 3 mm (P3) from the testing machine, and the static fracture load of only the zirconia abutment was evaluated.

The prepared test samples were fastened to each implant body at 35 N cm, the manufacturer’s recommended torque value. The implant body was fixed directly to the load tester (Electro Puls E3000, Instron, Norwood, MA, USA) using a collet chuck (EY Collet, Yukiwa Seiko, Niigata, Japan). After the upper part of the test sample was covered with a hemispherical load made of carbon tool steel material (SK material), a static load test was performed under the conditions of an inclination angle of 30°, room temperature (23 ± 1 °C), and a head speed of 0.5 mm/min (n = 3).

**Figure 3:**
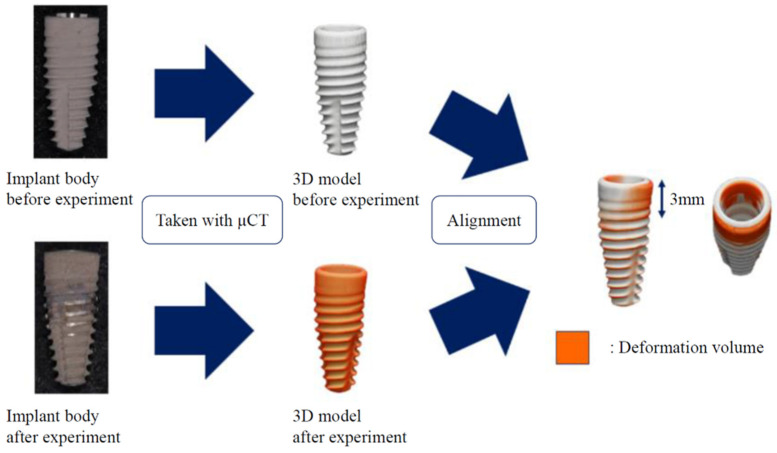
Flow chart for calculating the deformation volume. The sum of the protruding volume (mm^3^) was regarded as the amount of deformation.

In the P3 condition, which assumed the progress of bone resorption, the test body was placed with 3 mm of the platform exposed from the testing machine, and the static fracture load was evaluated for the abutment–implant body complex. There is the possibility that deformation of the implant body may occur under load because of platform exposure. Before and after the static load in the P3 condition, the implant body was imaged using micro-computed tomography (R_mCT, Rigaku, Tokyo, Japan) under a tube voltage of 90 kV, tube current of 160 µA, and voxel size of 40 µm. A 3D model was prepared for each implant. Additionally, the controls were only the implant body attached to the load tester (C-NB group, C-ST group). Before and after the experiment, the models were aligned with the analysis software (TRI / 3D-BON, RATOC, Tokyo, Japan) (Figure 3). The volume of the model protruding was compared before and after the experiment as the amount of deformation. One-way analysis of variance (ANOVA) and a Tukey’s test were used for statistical processing with the significance level set to 0.05.

**Figure 4:**
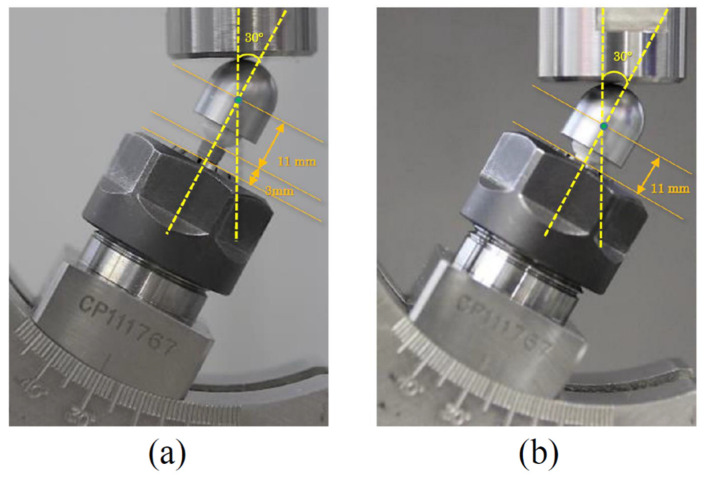
Installation of specimens with two different conditions. (**a**) P3: Specimen installed with 3 mm of the platform exposed. (**b**) P0: Specimen installed without exposure of the platform. Test conditions were modified according to ISO 14801:2016.

In the P0 condition, the static breaking load of the zirconia abutment alone was evaluated without exposure of the platform from the tester. Additionally, each component after the P0 condition was observed with a digital microscope (VHX-5000, Keyence, Osaka, Japan). The loading geometry is shown in Figure 4. The load value showing a peak on the static load–displacement curve was taken as the static fracture load. A student t-test was used for statistical processing of these breaking loads, and the significance level was set to 0.05. All statistical analyses were performed using R version 3.5.1 and Regression Modeling Strategies packages.

## 3. Results

**Figure 5:**
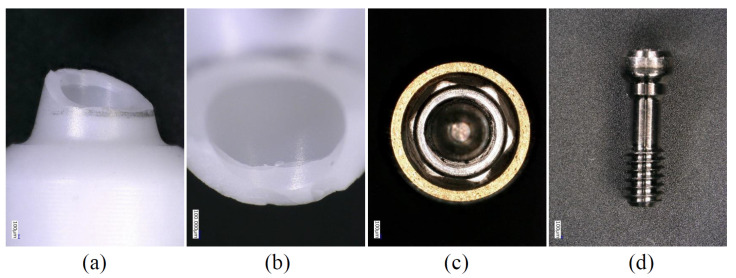
Enlarged images showing the components after the P0 condition (Z-NB group). (**a**) Fractured zirconia section, (**b**) Enlarged image of (**a**), (**c**) Implant body, (**d**) Abutment screw. There was no obvious deformation in (**c**) or (**d**).

**Figure 6:**
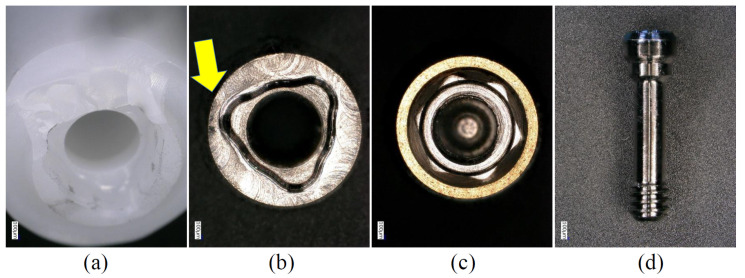
Enlarged images showing the components after the P0 condition (ZT-NB group). (**a**) Fractured zirconia section. (**b**) Titanium base; yellow arrow shows the area of deformation. (**c**) Implant body. (**d**) Abutment screw. There was no obvious deformation in (**c**) or (**d**).

**Figure 7:**
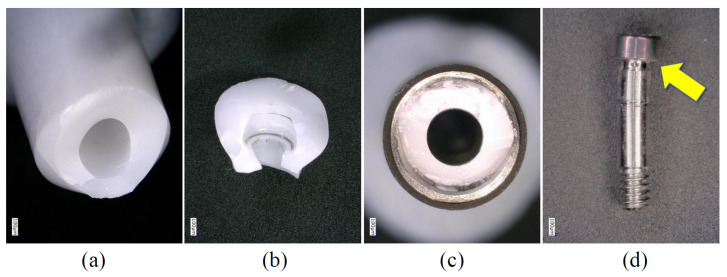
Enlarged images showing the components after the P0 condition (Z-ST group). (**a**) Fractured zirconia section. (**b**) Fractured fragment of zirconia. (**c**) Implant body; the fractured zirconia remained inside. (**d**) Abutment screw; yellow arrow indicates deformation at the top of the screw. There was no obvious deformation in (**c**).

**Figure 8:**
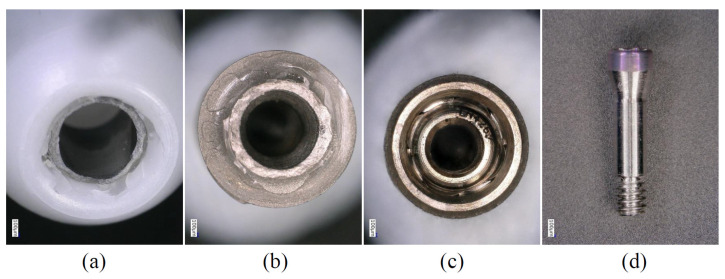
Enlarged images showing the components after the P0 condition (ZT-ST group). (**a**) Fractured section of the titanium base (zirconia side). (**b**) Fractured section of the titanium base (implant body side). (**c**) Implant body. (**d**) Abutment screw. There was no obvious deformation in (**c**) or (**d**).

In the P3 and P0 conditions, all specimens in the same group showed similar failure modes. In the Z-NB group, fracture of the zirconia was observed near the platform of the implant connection (Figure 5). In the ZT-NB group, fracture of the zirconia in the fitting part of the titanium base and deformation of the titanium base were observed (Figure 6). In the Z-ST group, as in the Z-NB group, fracture of the zirconia was observed near the platform (Figure 7). In the ZT-ST group, adhesion failure between the titanium base and zirconia, and failure of the titanium base itself were observed (Figure 8).

**Figure 9:**
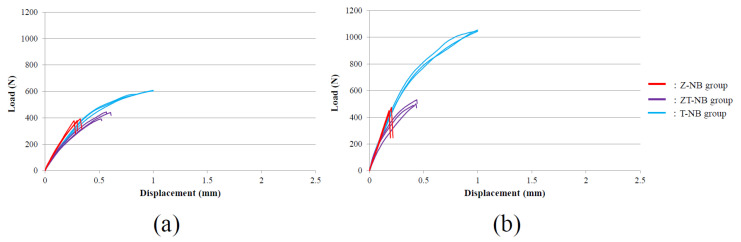
Load–displacement curves under static load showing the (**a**) P3 and (**b**) P0 conditions in the Z-NB, ZT-NB, and T-NB group specimens (n = 3). In the T-NB group, the load was stopped at a displacement of 1 mm.

The relationship between the compressive load and vertical displacement of the loaded part in the Z-NB, ZT-NB, and T-NB specimens is shown in Figure 9. In the P3 condition, the average load at failure was 383.8 ± 7.90 N in the Z-NB group and 425.6 ± 30.3 N in the ZT-NB group. In the P0 condition, the average load at failure was 459.9 ± 13.2 N in the Z-NB group and 507.3 ± 22.0 N in the ZT-NB group.

**Figure 10:**
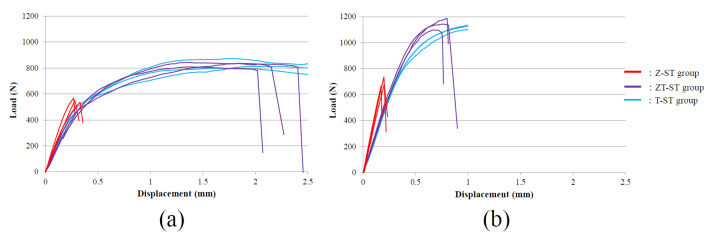
Load–displacement curve under static load showing the (**a**) P3 and (**b**) P0 conditions in the Z-ST, ZT-ST, and T-ST group specimens (n = 3). In the T-ST group in (**b**), the load was stopped at a displacement of 1 mm.

Figure 10 shows the relationship between the compressive load (N) in the Z-ST, ZT-ST, and T-ST specimens and the vertical displacement (mm) of the loaded part. In the P3 condition, the average load at failure was 551.2 ± 15.8 N in the Z-ST group and 827.9 ± 14.3 N in the ZT-ST group. In the P0 condition, the average load at failure was 693.9 ± 37.2 N in the Z-ST group and 1142.7 ± 36.9 N in the ZT-ST group.

In the P3 condition, in which the static fracture load of the abutment–implant body complex was evaluated, the fracture load of the titanium base type was significantly greater than the full zirconia base type in the ST groups (*p* < 0.001). No significant difference was observed in the NB groups (*p* = 0.082).

**Figure 11:**
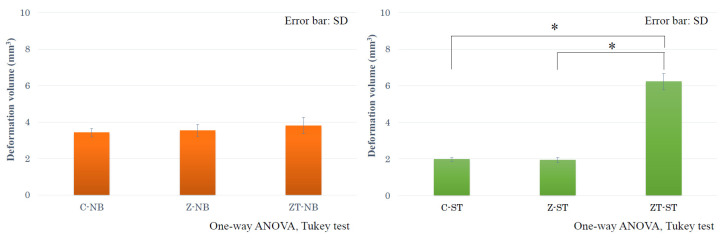
Deformation volume of the NB and ST groups. There was no significant difference between the three NB groups. The deformation volume of the ZT-ST group was significantly greater than the other two groups (*p* < 0.001).

The deformation volumes of the Z-NB and ZT-NB groups were 3.536 ± 0.327 mm^3^ and 3.803 ± 0.443 mm^3^, respectively. The deformation volume of the T-NB group averaged 3.420 ± 0.233 mm^3^, and there was no significant difference between all three groups. Alternatively, the deformation volumes of the Z-ST and ZT-ST groups were 1.94 ± 0.128 mm^3^ and 6.228 ± 0.447 mm^3^, respectively. The average deformation volume of the T-ST group was 1.973 ± 0.092 mm^3^, and the Z-ST group was not significantly different from the T-ST group. The deformation volume of the ZT-ST group was significantly greater than that of the other two groups (both *p* < 0.001) (Figure 11).

In the P0 condition, in which the static fracture strength of the zirconia abutment alone was evaluated, the fracture load of the titanium base type was significantly greater than that of the full zirconia type in both NB and ST groups (NB: *p* = 0.032, ST: *p* < 0.001).

When comparing the P3 and P0 conditions, the static fracture strength was smaller in the P3 condition than in all other specimens.

## 4. Discussion

Yttria partially stabilized tetragonal zirconia polycrystal (Y-TZP) has greater strength and mechanical properties than other zirconia-based ceramic materials [20,21,22,23]. Y-TZP is now used more frequently in clinical practice because it is esthetically pleasing. Many studies have reported that zirconia abutments do not detract from the color tone of soft tissue when compared with conventionally used titanium abutments [2,4,5,6,7,10,11,12,13,14,15].

The tapered conical connection implant, which has been frequently used in recent years, is advantageous in securing the thickness of the soft tissue around the implant body and preventing bone resorption and soft tissue retraction [16,17,18]. Therefore, combining a tapered joint type implant body and a zirconia abutment is considered effective for esthetic success.

Zirconia abutments are classified into two types: zirconia abutments and titanium base abutments [5]. Many clinical reports have cited mechanical problems, such as fracture, chipping, and detachment [15,24,25], for both types of zirconia abutments. The risks are exceptionally high when zirconia abutments are combined with a tapered conical connection implant [9,15], especially in narrow implants [25]. However, little research has been directed to the mechanical properties of zirconia abutments with taper joint bodies.

It is often reported that titanium-based zirconia abutments have significantly greater resistance to a bending moment than full zirconia abutment [2,6,7,8,9,10]. However, none of the reports used a uniform implant body [2,6,7,9,10] or CAD/CAM system [2,6,7,8,9,10] to prepare the specimens to allow for an accurate comparison. Therefore, factors other than the titanium base that may affect the mechanical properties could not be excluded. In this study, the actual mechanical properties were evaluated by examining NB and ST abutments with or without a titanium base for the same implant with a tapered conical connection. In addition, experiments were conducted with regular size abutments; however, in clinical practice, problems, such as fracture, have been reported with narrow abutments [25]. Cyclic loading tests on narrow abutments are necessary to investigate abutment designs for more severe conditions.

The room temperature was set to 23 ± 1 °C, which is often adopted as the standard condition in laboratories. When conducting experiments that simulate intraoral conditions, such as cyclic loading tests, it is necessary to use temperatures and humidity that are closer to that of actual patients.

In all samples, fracture occurred at the interface between the implant body and zirconia or between the zirconia and titanium base (Figure 5, Figure 6, Figure 7 and Figure 8). After testing, deformation was also observed in the titanium base in the ZT-NB group and in the screw head in the Z-ST group. This was thought to be the result of stress concentration at the interface between the implant components and at the site where deformation was observed.

P0 experiments rejected the null hypothesis that the two types of abutments would have similar fracture loads. The static fracture load of the titanium base abutment was significantly greater than the full zirconia abutment, possibly because of the increased resistance to compressive and tensile stresses near the platform caused by the titanium base [26].

In both P3 and P0 conditions, the load–displacement curve of the ZT-ST group in the low load region showed similar behavior with a T-ST group. The cemented zirconia abutment was an example of an integrated titanium base and zirconia with mechanical properties similar to those of a titanium abutment.

Additionally, the breaking load of the ZT-ST specimens greatly exceeded that of the ZT-NB specimens. Given that the height of the titanium base can affect the failure load of a zirconia abutment [27], differences in the titanium base design of different manufacturers may be related to the static fracture load of the zirconia abutments. The purpose of this study was not to statistically compare the values between manufacturers, which differ in the shape of the connection of the implant body. The relationship between different titanium-based designs and abutment fracture resistance should be examined in another study.

Comparison of the P3 and P0 conditions revealed that the static fracture strength decreased when the height of the implant component increased. This decreased fracture strength is related to the fact that immense stress was applied to the weakest part, even at relatively low loads, by the bending moment [27]. Clinically, if bone resorption progresses and the height of the implant component on the bone edge increases, it is likely to be mechanically disadvantageous. The implant would then be prone to problems such as breaking of the abutment. This result was similar to that of a previous study that found that the vertical height of the implant superstructure relates to the long-term prognosis of the abutment, the risks of abutment fracture increases, especially at a height of 14 mm or greater [15]. There are also studies reporting the relationship between abutment height and marginal bone resorption around the implants [28,29,30]. The effect of this height was not verified in this study and needs to be further investigated.

One of the limitations of this study was that the number of samples was small (n = 3). A post hoc test to determine the adequacy of the sample size was conducted using a power analysis program (G*Power) with the fracture load of each group. In ST, both the P3 and P0 conditions showed a power of 1.0, indicating a significant number of samples. For NB, the power was 0.42 for the P3 condition and 0.67 for the P0 condition. The sample size for NB was small, and it was highly likely that a Type 2 error occurred. To obtain statistically reliable results, experiments should be repeated with more samples, especially in NB.

One of the features of this study was that the presence and amount of deformation of the implant body before and after the P3 experiment could be evaluated in 3D. However, the obtained 3D model can be affected by artifacts. Since each sample was taken independently and under the same conditions, the effect of artifacts and the difference between them was considered to be very small. However, the numerical values of the volume regarded as the amount of deformation in this study were not reliable. Overall, the null hypothesis that there was no difference in the amount of deformation of the implant body after the test for the two types of abutments was rejected at ST. The results of the Z-NB and Z-ST groups suggest that the full zirconia abutment was destroyed only inside the zirconia abutment; other components were hardly affected.

Additionally, from the results of the ZT-NB group, the shape of the titanium base unique to the NB limits damage to the implant body even when the zirconia abutment was destroyed. Alternatively, the results of the ZT-ST group indicate that the abutment could cause damage to the implant body instead of breaking, even under a heavy load, because of the titanium-based design. However, in this experiment, only the appearance of the implant body after loading that significantly exceeds the average occlusal force of an adult male [31] was confirmed. In other words, it is not clear how much load was causing the deformation. As a limitation of this study, it is impossible to state that the result is directly linked to the actual clinical condition because the mechanical evaluation by cyclic loading was not performed. However, the high fracture resistance of the abutment and the fact that the implant body was not damaged are both critical for the long-term prognosis of implant treatments.

Combined with the P3 results, the design of the titanium base can be enhanced to ensure that the strength of the zirconia abutment is similar to that of the ST type. It may also be possible to make a fail-safe design that does not damage the implant body at the time of zirconia abutment destruction, as in the NB type.

## 5. Conclusions

In this study, the mechanical properties were examined related to the presence of a titanium base in a zirconia abutment and the fracture resistance in a tapered conical connection implant. The following conclusions were obtained under the limitation of the insufficient number of samples and static load test.

- The presence of a titanium base could increase the fracture resistance of zirconia abutments.
- The titanium base abutment showed higher fracture resistance than the full zirconia abutment but might damage the implant body.
- The full zirconia abutment showed lower fracture resistance than the titanium base abutment but is less likely to damage the implant body.

Clinicians should select and design zirconia abutments after considering the characteristics mentioned above.

## Data Availability

Data sharing not applicable.

## Abbreviations

CAD/CAM: computer-aided design/computer-aided manufacturing; NB: Nobel Biocare; ST: Straumann.
